# Acute interstitial nephritis due to acute pyelonephritis in a young woman: diagnostic utility of CK7 and CD10 immunostaining

**DOI:** 10.1007/s13730-026-01115-4

**Published:** 2026-04-29

**Authors:** Naoto Eriguchi, Keiki Shimada, Daisuke Katagiri, Hitomi Otani, Yuri Katayama, Kanako Terakawa, Mikako Koizumi, Harui Bamba, Tomonobu Kajio, Mariko Kawamura, Minami Suzuki, Hideki Miyazaki, Hideki Takano

**Affiliations:** 1 Department of Nephrology, National Center for Global Health and Medicine, Japan Institute for Health Security, 1-21-1, Toyama, Shinjuku-Ku, Tokyo, 168-8655 Japan; 2 Department of Pathology, National Center for Global Health and Medicine, Japan Institute for Health Security, 1-21-1, Toyama, Shinjuku-Ku, Tokyo, 168-8655 Japan

**Keywords:** Acute pyelonephritis, Acute interstitial nephritis, Renal biopsy, Cytokeratin 7, Cluster of differentiation 10

## Abstract

Acute Interstitial Nephritis (AIN) is a common cause of acute kidney injury (AKI) and may lead to irreversible renal scarring if the diagnosis is delayed. Although most cases are drug-induced, AIN can also occur in association with acute pyelonephritis (APN). Nevertheless, given that antibiotics are routinely administered for APN, cases of APN-associated AIN may be erroneously classified as drug-induced AIN. Distinguishing these two entities histologically remains challenging; however, their therapeutic strategies differ. We describe a case involving a previously healthy 21-year-old woman who presented with a fever and rapidly progressive renal impairment. Laboratory evaluation revealed serum creatinine and C-reactive protein concentrations of 3.54 mg/dL and 25.06 mg/dL, respectively, prompting an urgent renal biopsy. Histopathological examination by light microscopy demonstrated a diffuse interstitial and tubular inflammatory infiltrate predominantly composed of neutrophils, consistent with AIN. Based on these pathological findings and the clinical course, AIN secondary to APN was suspected. The patient was managed with an intensified antibiotic regimen without corticosteroid therapy, resulting in complete renal recovery. To further refine the diagnosis after hospital discharge, we examined immunostaining patterns across tubular segments. Immunostaining demonstrated a neutrophil-predominant infiltrate in the distal tubules and collecting ducts, which showed a segmental pattern characteristic of APN-related injury. This case highlights the potential utility of immunostaining in distinguishing APN-associated AIN from drug-induced AIN and supports its incorporation into diagnostic evaluation.

## Introduction

Acute Interstitial Nephritis (AIN) is characterized by inflammatory infiltration of the renal tubules and interstitium. This process results in tubulitis and edema and may progress to interstitial fibrosis [1]. Drug exposure accounts for 60–70% of AIN cases [2]. AIN can also be induced by acute pyelonephritis (APN), although it is rarely reported [3]. APN is a community-acquired bacterial infection that typically presents with systemic symptoms, including fever [4]. Severe AKI associated with APN usually reflects sepsis-associated hypotension and acute tubular necrosis, which is particularly prominent in older adults and patients with chronic kidney disease [5]. APN-induced severe AKI is less common in the absence of coexisting urinary tract obstruction [6].

Clinicians routinely prescribe antibiotics for APN, and these drugs are a major cause of drug-induced AIN. Consequently, distinguishing APN-related AIN from drug-induced AIN based solely on clinical presentation is challenging. Moreover, diagnostic ancillary tests, such as drug-induced lymphocyte stimulation test (DLST), have limited sensitivity and are difficult to interpret [7]. Notably, immunostaining may allow for clearer differentiation. Specifically, proximal tubules express cluster of differentiation 10 (CD10), whereas distal tubules and collecting ducts express cytokeratin 7 (CK7) [8–10]. Early drug-induced AIN typically involves the proximal tubules, and APN typically exhibits neutrophilic infiltration of the distal tubules and collecting ducts [11, 12]. Therefore, drug-induced AIN demonstrates CD10-positive proximal tubular involvement, whereas APN-induced AIN exhibits CK7-positive distal tubular involvement. We used segment-specific markers to corroborate our suspicion of APN-associated AIN in a young woman. Her biopsy demonstrated neutrophil-predominant inflammation confined to the distal nephron segments, underscoring the utility of this technique for differential diagnosis. Early recognition of this pattern is important, as APN-related AIN responds to targeted antimicrobial therapy rather than empirical steroid administration. Adoption of segment-specific immunohistochemical panels for renal biopsy evaluation may shorten the time to diagnosis, prevent unnecessary immunosuppression, and improve renal outcomes in affected patients.

## Case report

A 21-year-old woman developed a high-grade fever, which persisted for 6 days prior to admission. During this period, she self-administered acetaminophen, but her symptoms did not improve. 2 days before admission, she visited a local clinic, where she was prescribed levofloxacin hydrate and loxoprofen sodium. Laboratory evaluation at that time revealed a markedly elevated C-reactive protein level of 34 mg/dL and serum creatinine (sCre) level of 3.72 mg/dL. Owing to these abnormal findings, she was referred to our department and subsequently admitted on hospital day 0 for further evaluation and management. On admission, her body mass index was 17.6. Her vital signs included a temperature of 38.0 °C, pulse rate of 117 bpm, blood pressure of 112/72 mmHg, and respiratory rate of 18 breaths/min. Physical examination revealed no nuchal rigidity or jolt accentuation. Conjunctival injection and jaundice were absent. Cervical lymphadenopathy was not detected; however, pharyngeal erythema was observed. No peripheral edema or skin rash was observed.

The results of urinalysis and blood tests are summarized in Table. Urinalysis demonstrated proteinuria, glycosuria, bacteriuria, and pyuria, along with marked elevation of tubular injury markers, including urinary β2-microglobulin (u-β2MG) and liver-type fatty acid–binding protein (L-FABP), indicating significant interstitial involvement. [13–15].

Blood tests revealed severe acute kidney injury (AKI) accompanied by a pronounced systemic inflammatory response. Additional immunological testing showed elevated total Immunoglobulin E (IgE), while antinuclear and anti-neutrophil cytoplasmic antibodies were negative, with no disease-specific immunological abnormalities identified. Urinalysis demonstrated pyuria and bacteriuria, consistent with urinary tract infection (Table 1).

**Table 1:** Laboratory findings on admission

	On admission	On discharge	Normal range
White blood cells (× 10^3^/μL)	19.79	4.79	3.30–8.60
Neutrophils (%)	89	63.2	–
Eosinophils (%)	0.0	3.3	–
Hemoglobin (g/dL)	12.7	11.9	11.6–14.8
Platelet (× 10^4^/μL)	10.4	53.0	15.8–34.8
Total Protein (g/dL)	6.0	7.0	6.6–8.1
Albumin (g/dL)	2.5	3.4	4.1–5.1
Blood urea nitrogen (mg/dL)	84.1	12.4	8.0–20.0
Serum creatinine (mg/dL)	3.54	0.76	0.46–0.79
C-reactive protein (mg/dL)	25.06	0.09	< 0.14
Aspartate aminotransferase (U/L)	31	25	13–30
Alanine aminotransferase (U/L)	26	21	7–23
Sodium (mEq/L)	132	141	138–145
Potassium (mEq/L)	3.4	4.3	3.6–4.8
Chloride (mEq/L)	96	105	101–108
Calcium (mg/dL)	8.4	9.5	8.8–10.1
Total bilirubin (mg/dL)	1.2	0.7	0.4–1.5
Prothrombin time-INR	1.05	1.05	0.9–1.10
Activated partial thromboplastin Time (s)	35	33	25–35
Hematuria	3 +	Negative	Negative
Proteinuria	1 +	Negative	Negative
Urinary L-type fatty acid–binding protein (μg/gCr)	28.2	–	< 8.4
Urinary β2-microglobulin (μg/L)	19,528	–	30–340
Urinary leukocytes (/HPF)	30–49	1–4	< 10
Urinary erythrocytes (/HPF)	1–4	Negative	< 5
Immunoglobulin G (mg/dL)	755	–	861–1747
Immunoglobulin A (mg/dL)	182	–	93–393
Immunoglobulin M (mg/dL)	178	–	50–269
Complement 3 (mg/dL)	109	–	73.0–138
Complement 4 (mg/dL)	21.5	–	11.0–31.0
total immunoglobulin E (U/mL)	1245	–	< 232
Rheumatoid factor (IU/mL)	17.8	–	< 15
MPO-ANCA (IU/mL)	< 0.2	–	0.0–3.4
PR3-ANCA (IU/mL)	0.7	–	0.0–1.9
Antistreptolysin-O (IU/mL)	16	–	0–40
Serum β2-microglobulin (mg/L)	13.3	–	< 2.0

ANCA: Anti-neutrophil cytoplasmic antibody

Urine and blood cultures were obtained at the time of admission; however, all cultures remained negative. Antibiotic therapy had already been initiated prior to or at the time of culture collection, which may have contributed to the negative results.

**Fig. 1:**
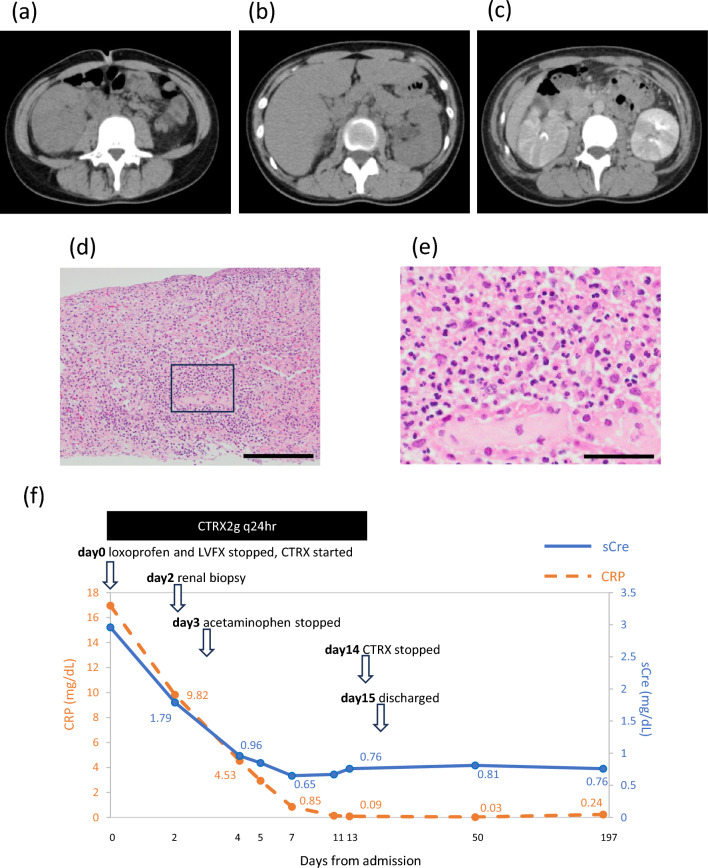
CT findings, renal recovery timeline, and renal biopsy findings. **a**, **b** Non-contrast CT of the abdomen. The bilateral kidneys are enlarged. **c** Contrast-enhanced CT demonstrate bilateral renal enlargement with right-sided predominance, multiple poorly enhanced areas within the renal parenchyma, and increased perirenal fat stranding. **d**, **e** Renal biopsy showing neutrophil-predominant infiltration of the tubules and interstitium, with scant eosinophils. **d** Lower magnification (Hematoxylin–Eosin staining × 200, Scale bar = 100 µm); **e** Higher magnification (Hematoxylin–Eosin staining × 400, Scale bar = 50 µm). **f** Clinical course of serum creatinine and C-reactive protein levels during hospitalization and follow-up. The x-axis represents time in days plotted on a logarithmic (log10) scale. Renal biopsy was performed the day 2. With the change of antibacterial drug and discontinuation of the medications suspected to have caused renal dysfunction, renal function and inflammatory response returned to the normal range on day 9, and the antibacterial drug was discontinued on day 14. After discharge on hospital day 15, during follow-up, renal function remained stable Computed tomography: CT; C-reactive protein: CRP; serum creatinine: sCre; levofloxacin: LVFX; ceftriaxone: CTRX

Moreover, non-contrast computed tomography (CT) was performed to avoid contrast exposure in the setting of severe AKI (Fig. 1a, b). According to the radiology report, the non-contrast CT demonstrated bilateral renal enlargement with right-sided predominance, increased perirenal fat stranding, and mild dilation of the left renal pelvis, findings that were not inconsistent with APN. As renal function subsequently showed an improving trend, contrast-enhanced CT was performed on hospital day 4. The contrast-enhanced CT demonstrated bilateral renal enlargement with right-sided predominance, multiple poorly enhanced areas, and increased perirenal fat stranding, findings that were not inconsistent with APN (Fig. 1c).

A renal biopsy was performed on day 2. Histopathological analysis identified 18 glomeruli without evidence of sclerosis, crescent formation, or adhesions. Hematoxylin and eosin (HE) staining revealed WBC casts within tubules and infiltration of inflammatory cells (predominantly neutrophils) into the interstitial compartment (Fig. 1d, e). We perfomed the DLST at day 8. The DLST was positive for acetaminophen.

Given the presence of pharyngitis, post-streptococcal glomerulonephritis was initially considered. Furthermore, elevated serum total IgE levels and a positive DLST for acetaminophen suggested the possibility of drug-induced AIN. However, renal biopsy demonstrated infiltration of neutrophil-predominant inflammatory cells. In conjunction with the presence of pyuria and bacteriuria, the final diagnosis was AIN secondary to APN.

On admission, as an infectious etiology for a fever could not be excluded, empirical antibiotic therapy was continued. However, given the possibility of drug-induced AIN, levofloxacin was switched to ceftriaxone, and loxoprofen was discontinued. Acetaminophen was also discontinued on day 3.

Renal function showed an improving trend, with u-β2MG improving to 111 μg/L and sCre to 0.65 mg/dL by day 7 of hospitalization. Furthermore, by day 13 of hospitalization, sCre levels had improved to 0.76 mg/dL and C-reactive protein levels to 0.09 mg/dL. Antibiotic therapy was discontinued on day 14, and the patient was discharged on day 15. After discharge on hospital day 15, the patient was followed in the outpatient clinic without any additional treatment. During follow-up, renal function remained stable, and no recurrence of abnormal urinalysis findings, such as proteinuria or hematuria, was observed. There was no clinical evidence of relapse or progression, indicating sustained recovery. (Fig. 1f).

The differential diagnosis in this case included APN and drug-induced AIN, with APN being considered the primary cause of interstitial injury. However, the DLST results suggested the possibility of drug-induced AIN. After discharge from the hospital, she also recognized the potential value of differentiating diagnoses based on the site of tubular injury to further support the diagnosis. Drug-induced AIN predominantly affects the proximal tubules, whereas APN primarily involves the distal tubules [7, 8]. Therefore, we performed additional immunostaining for CD10 and CK7 to localize the injury site.

Immunostaining for CK7 and CD10 was performed using the BenchMark ULTRA automated staining system (Roche Diagnostics, Indianapolis, IN, USA). All reagents and antibodies were obtained from Roche Diagnostics. Formalin-fixed, paraffin-embedded tissue sections were deparaffinized at 72 °C, followed by pretreatment with Pretreatment 1 for 12 min. Antigen retrieval was carried out using Ultra Cell Conditioning 1 (CC1). For CK7 staining, cell conditioning was performed at 95 °C for 64 min, and sections were incubated with anti-CK7 antibody (clone SP52) for 32 min. For CD10 staining, cell conditioning was performed at 100 °C for 64 min, followed by a pre-primary peroxidase inhibition step and incubation with anti-CD10 antibody (clone SP67) for 32 min. After antibody incubation, sections were washed using the UltraWash solution. Counterstaining was performed with Hematoxylin II for 16 min, followed by Bluing Reagent for 4 min.

**Fig. 2:**
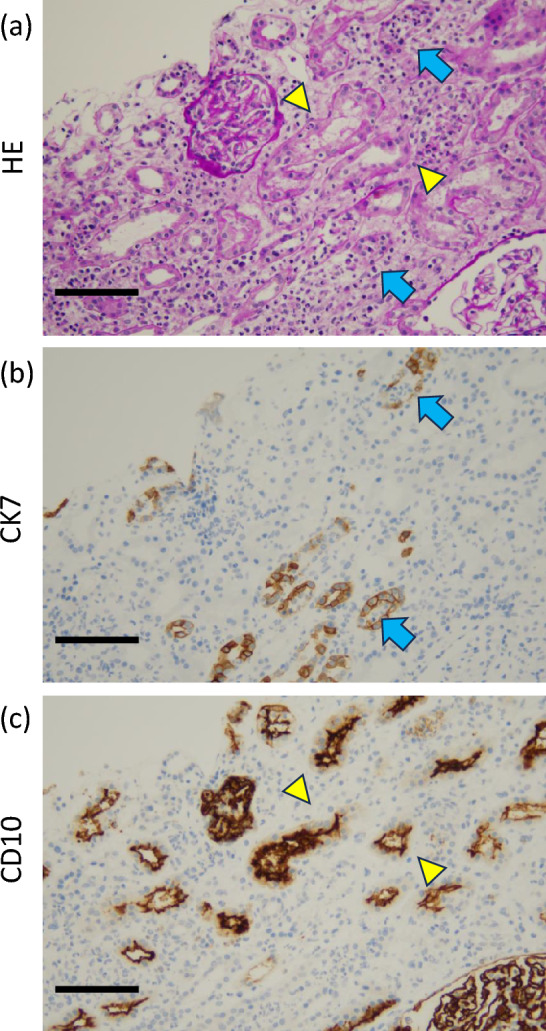
Histological and immunohistochemical features of interstitial inflammation. **a** Tubules positive for CK7 (blue arrows) exhibited greater infiltration of inflammatory cells than tubules positive for CD10 (yellow triangle). (Hematoxylin–Eosin stain × 400, Scale bar = 100 μm) **b** CK7 immunostaining shows positive staining in distal tubules, where inflammatory cell infiltration and structural destruction are predominantly observed (blue arrows).(CK7 immunostaining × 400, Scale bar = 100 µm) **c** CD10 immunostaining shows positive staining in proximal tubules (yellow triangle), whereas minimal staining is present in tubules with predominant inflammatory cell infiltration. (CD10 immunostaining × 400, Scale bar = 100 µm). Cytokeratin7: CK7; Cluster of differentiation10: CD10

Renal pathologist who was blinded to the clinical hypothesis regarding the etiology of AIN evaluated this tissue. Under this blinded assessment, the pathologist independently confirmed that in tubules positive for CK7, HE staining showed inflammatory cell infiltration (Fig. 2a, b: blue arrows), while in tubules positive for CD10, inflammatory cell infiltration was less prominent in HE staining (Fig. 2a, c: yellow arrows).

To evaluate semi-quantitative assessment of inflammatory distribution, image analysis was also performed using ImageJ software (National Institutes of Health, Bethesda, MD, USA). Eight representative immunostained images were selected for analysis. Regions of interest (ROIs) corresponding to CK7-positive and CD10-positive tubular segments were manually delineated on each image, and the relative area fractions were calculated. Using this approach, the mean relative area fraction of CD10-positive tubular segments was 11.8%, whereas that of CK7-positive tubular segments was 5.9%.

Immunostaining results further supported the possibility of AIN associated with APN, as inflammatory infiltrates were predominantly observed in CK7-positive tubular segments rather than CD10-positive segments.

## Discussion

This case provides two key insights. First, it demonstrates that neutrophilic inflammation associated with APN can directly cause AIN. Second, the immunohistochemical localization patterns of CK7 and CD10 offer a useful diagnostic tool to differentiate the etiology of AIN, with direct applicability in clinical practice.

Our first major finding is that neutrophilic inflammation in the context of APN can indeed lead to AIN. Previous histopathological studies of APN-induced AKI have described interstitial nephritis characterized by scattered neutrophil infiltrates, tubular necrosis, and pustule formation [3]. These studies have also reported bilateral renal enlargement, sterile pyuria, and, in some cases, the requirement for renal replacement therapy. Progression to sepsis was not suspected as the patient was not hypotensive and blood tests revealed no evidence of elevated liver function enzymes or hematopoietic compromise. However, a renal biopsy of this patient revealed a dense interstitial neutrophilic infiltrate and tubular damage consistent with infectious AIN. Neutrophils were preferentially present in CK7-positive distal tubules, consistent with inflammation caused by APN. Therefore, neutrophilic inflammation associated with APN was confirmed to cause AIN in this case. Clinicians have attributed most AIN to drug-induced hypersensitivity [2]. However, the pathogenesis of APN-induced AIN likely involves direct bacterial injury and tubular damage associated with neutrophil infiltration [12]. These findings indicate that APN may independently cause histological features of AIN beyond those related to hemodynamics and prerenal AKI.

Another important finding is the diagnostic value of immunostaining in AIN. Pathologists routinely use immunostaining to classify subtypes of renal cell carcinoma, including clear cell and papillary carcinoma [16]. However, CK7 and CD10 have different expression patterns in different tubular sites, with distal tubules being CK7 positive and proximal tubules being CD10 positive [8–10]. In the present case, immunostaining demonstrated that neutrophils were predominantly located in CK7-positive distal tubules, whereas they were less frequently observed in CD10-positive proximal tubules. This distal predominance pattern is consistent with the laboratory and clinical findings of APN-induced AIN [6]. While immunostaining indicated a distal/collecting duct predominance (CK7-positive segments), u-β2MG—a clinical marker of proximal tubular dysfunction—was markedly elevated in the early phase but declined to 111 μg/L by hospital day 7, in parallel with improvement in renal function (sCre 0.65 mg/dL) on day 7 and C-reactive protein 0.09 mg/dL by day 13. u-β2MG is also known to increase in association with inflammatory cytokine activity, reflecting immune activation rather than isolated proximal tubular injury[17, 18]. Because biomarker sampling and renal biopsy were not performed simultaneously, and because active interstitial inflammation may involve overlapping tubular segments, we interpret u-β2MG as supportive clinical context rather than a definitive indicator of the primary site of injury. In this case, although we did not evaluate other immune molecular markers, drug-induced AIN is often characterized by lymphocytic infiltration [1, 2], and our histopathological examination confirmed a neutrophilic pattern in APN-induced AIN, which supports an infectious etiology [12].

In summary, drug-induced AIN commonly shows lymphocytic infiltration of CD10-positive proximal tubules [2], whereas APN-induced AIN commonly shows neutrophilic infiltration of CK7-positive distal tubules. Therefore, based on the neutrophilic infiltration of the CK7-positive distal tubules, we conclude that APN-induced AIN is more suspicious in this case.

However, biopsy alone cannot completely exclude drug-induced AIN. As the patient had recently used loxoprofen and acetaminophen, we performed DLST to evaluate for drug-induced AIN [7]. The results were positive for acetaminophen; however, the sensitivity of DLST ranges from 30 to 80%, and specificity is often less than 90% [19]. Infection can also alter cytokine profiles and T-cell activation, leading to false-positive or false-negative DLST [20, 21]. We performed the DLST on hospital day 8, during the early recovery phase. Given the limited diagnostic accuracy of DLST and the possibility of false-positive results in the setting of active or recent infection, the positive result for acetaminophen was interpreted as supportive but not definitive.

Overall, the clinicopathological findings in this case were consistent with infection-associated AIN, while a drug-related component could not be completely excluded. Histological analysis demonstrated a dense neutrophilic infiltrate predominantly localized to CK7-positive distal tubules, a pattern that supports an infectious etiology. Considering the anatomic distribution of inflammation, persistent urinary findings, and the limitations of DLST during infection, APN was considered the plausible underlying cause of AIN in this patient.

However, to avoid potential nephrotoxicity or allergic reactions, both acetaminophen and loxoprofen were discontinued. Although corticosteroids are commonly used to treat AIN [22], we continued the antibiotic therapy, which gradually improved the inflammatory markers and renal function of the patient.

In summary, we demonstrated that neutrophilic inflammation from APN can mimic or induce AIN, and immunostaining patterns, specifically CK7/CD10, can meaningfully inform clinical decision-making. Recognizing APN as a cause of AIN may prevent misdiagnosis of drug hypersensitivity and avoid unnecessary immunosuppression. Our findings highlight immunostaining as a practical tool to anatomically characterize AIN and guide targeted therapy. Broader adoption and systematic evaluation of such staining techniques may enhance diagnostic accuracy in challenging cases of AIN.
